# Assessment of the Left Atrial Reservoir Function and Left Atrial Volume After Percutaneous Balloon Mitral Valvuloplasty Using Peak Atrial Longitudinal Strain

**DOI:** 10.7759/cureus.22395

**Published:** 2022-02-19

**Authors:** Siddharth Samrat, Najeeb U Sofi, Puneet Aggarwal, Santosh K Sinha, Umeshwar Pandey, Awadhesh K Sharma, Mahmodullah Razi, Mohit Sachan, Praveen Shukla, Ramesh Thakur

**Affiliations:** 1 Cardiology, Laxmipat Singhania Institute of Cardiology, Kanpur, IND; 2 Cardiology, Atal Bihari Vajpayee Institute of Medical Sciences and Dr Ram Manohar Lohia Hospital, Delhi, IND

**Keywords:** atrial fibrillation, percutaneous balloon mitral valvulotomy, strain rate imaging, peak atrial longitudinal strain, left atrial reservoir function, mitral stenosis

## Abstract

Objective

To evaluate the impact of successful percutaneous balloon mitral valvuloplasty (BMV) on left atrial (LA) reservoir function and LA volume in patients with severe mitral stenosis (MS) using peak atrial longitudinal strain (PALS).

Method

This was a prospective, non-randomized observational study conducted at the Laxmipat Singhania (LPS) Institute of Cardiology, Kanpur from August 2018 to February 2020 among patients with severe rheumatic MS undergoing BMV to assess LA reservoir function and its volume after BMV using PALS. Inclusion criteria were symptomatic severe rheumatic MS (NYHA ≥II), normal ventricular systolic function, and suitable valve morphology. Exclusion criteria were the coexistence of aortic valve involvement, left atrial appendage clot, mitral leak more than mild, pregnancy, hypertension, diabetes, and coronary artery disease. To assess LA reservoir function and its volume after BMV, PALS was used. LA was divided into six regions of interest and longitudinal strain curves of individual segments together with global strain were recorded. PALS was calculated at baseline 24 hours following the intervention, and at three months of follow-up.

Result

Successful BMV was performed in 260 patients (109 or 41.9% males and 151 or 58.1% females), resulting in significant improvement in mitral valve area (MVA) (0.89±0.11 cm^2^ vs. 1.83±0.3 cm^2^; p<0.001). The mean age of patients was 26.7±4.7 years; 214 (82.3%) patients were in normal sinus rhythm (NSR) while 46 (17.7%) had atrial fibrillation (AF).

Significant improvement in PALS was noted immediately following the procedure (6.5±11.6% vs. 7.7±10.5%; p< 0.001) and it continued to improve at three months of follow-up (6.5±11.6% vs. 11.3±12.5%; p<0.001), which was 24% and 74% improvement from baseline respectively. Significant reduction in indexed left atrial (LA) volume was observed immediately following the procedure (56.8±14.3 ml/m^2 ^vs 48.4±12.5 ml/m^2^; p=0.003), and at three months of follow-up (56.8±14.3 ml/m^2^ vs. 45.4±13.3 ml/m^2^; p=0.002). Those with AF had lesser improvement in PALS in comparison to those with NSR (60% vs. 84%; p=0.044) at three months of follow-up. At three months, the increase in PALS was also lower in patients with a history of stroke as compared to those without it (55% vs 80%; p=0.039). Both LA volume and indexed LA volume reduced significantly immediately at 24 hours and during follow-up.

Conclusion

LA reservoir function, as assessed by PALS, is reduced in patients with severe MS. It improved significantly within 24 hours following BMV and continued to improve at three months of follow-up. It is an underutilized modality among patients of MS for decision-making prior to intervention and to assess the effect of the intervention.

## Introduction

The left atrium (LA) acts as a reservoir, conduit, and pump [1,2]. It acts as a reservoir during left ventricular systole (LV) when the mitral valve is closed, LA is relaxed, and its annulus is temporarily displaced toward the apex [3]. It acts as a conduit in diastole when blood moves forward through the open mitral valve and as a pump to augment left ventricular preload at the end of the diastole [4].

In patients with mitral stenosis (MS), the function of the LA gets disrupted because of increased afterload, which leads to its enlargement and pressure overload which further gets aggravated with the onset of atrial fibrillation as its pump function completely ceases [4-7]. With long-standing MS, there is disorganization of the atrial muscle fiber, which subsequently leads to fibrosis [8]. Assessment of atrial function using atrial volume is an operator and load‑dependent process, thus lacking accuracy. The LA reservoir function can be better assessed by peak atrial longitudinal strain (PALS) using strain imaging. LA strain measurements are performed noninvasively using tissue Doppler imaging (TDI), speckle tracking echocardiography (STE), and velocity vector imaging (VVI) based on longitudinal strain and strain rate curves. In the reservoir phase, LA fills and stretches, leading to positive atrial strain which decreases when the mitral valve opens, causing negative deflection of the strain curve, and reaches a plateau analogous to diastasis and second deflection in the strain curve which corresponds to atrial systole. PALS is measured at the end of the reservoir phase while peak atrial contraction strain (PACS) corresponds to active atrial contraction. In patients with mitral stenosis, impairment of PALS has been reported in asymptomatic patients and is a predictor of atrial fibrillation (AF) and cardiovascular events [9]. It is also one of the most powerful predictors of new-onset AF on follow-up, progression from paroxysmal to persistent AF [9,10], and often precedes LA enlargement [11,12]. Successful percutaneous balloon mitral valvuloplasty (BMV) leads to early improvement in left atrial reservoir and conduit functions, without significantly affecting left atrial pump function [13]. We sought to evaluate the impact of successful BMV on the left atrial reservoir function and volume in patients with severe mitral stenosis using PALS. As many patients with rheumatic MS are in AF, we included patients with AF in our study. We also followed patients for three months to evaluate the long-lasting effects of BMV in mitral stenosis patients.

## Materials and methods

This was a prospective, non-randomized observational study, which was conducted at the Laxmipat Singhania Institute of Cardiology (LPSIC), GSVM Medical College, Kanpur from August 2018 to February 2020 among patients with severe rheumatic MS to assess LA reservoir function and its volume after BMV using PALS. The study was approved by the LPSIC ethical committee. The inclusion criteria were symptomatic severe rheumatic MS (NYHA ≥II), normal left ventricular systolic function (ejection fraction ≥55%), right ventricular systolic function (annular velocity ≥9.5 cm/s), and suitable valve morphology by echocardiography (presence of dark zone on commissures). Exclusion criteria were coexistence of aortic valve involvement, poor echo window, left atrial appendage clot, moderate or severe mitral regurgitation, pregnancy, hypertension, diabetes, and coronary artery disease. All patients were subjected to detailed clinical evaluation, including an electrocardiogram, weight, height, and transthoracic echocardiography using Vivid6 GE echocardiography machine (GE Healthcare, Chicago, Illinois). All measurements of ventricular parameters (systolic and diastolic dimension, interventricular septal thickness, posterior wall diameter) were recorded using the leading-edge technique as per recommendations laid down by the American Society of Echocardiography [14]. The severity of mitral stenosis was categorized using the mitral valve area (MVA) by planimetry, pressure half time (PHT), and mean transmitral gradient using continuous-wave Doppler [15]. Pulmonary artery systolic pressure was calculated using tricuspid regurgitation velocity and right atrial pressure based on inferior vena cava diameter. All these parameters were taken at baseline, 24 hours following BMV, and at three months of follow-up. Written informed consent was obtained from all patients or legal guardians in case of a minor. LA size and volume (maximum during ventricular systole and smallest during ventricular late diastole) were measured in apical four-chamber view [16].

Atrial strain imaging

**Figure 1:**
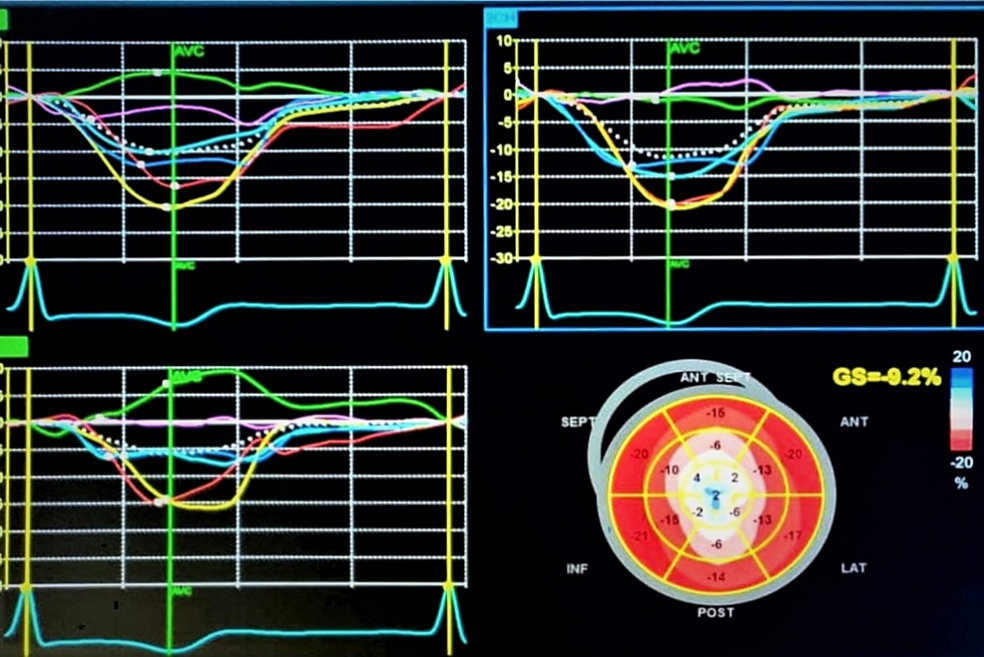
Left atrial strain of six segments as regions of interest depicted by different colours and overall PALS shown by white dots (A) Apical 4 chamber view; (B) Apical 2 chamber; (C) overall LA strain of all views where PALS is calculated at the end of T wave; (D) Bulls' eye representing various LA strain PALS: peak atrial longitudinal strain; LA: left atrium

LA regional function and deformation properties were studied using 2D speckle strain imaging. Apical four and two-chamber views of LA were obtained at relatively high frame rates (60-80 fps). Region of interest (ROI) was adjusted following tracing its endocardial border in both views. In regions of discontinuities (pulmonary veins and LA appendage), extrapolation of LA endocardial and epicardial surfaces at the junction of these structures was performed to obtain the ROI. The ROI was divided into six segments and a total of 12 segments were analyzed with the software generating the individual segmental longitudinal strain curves together with global strain in each view (Figure 1). PALS was calculated at baseline, 24 hours following intervention as well as at the three-month follow-up.

Percutaneous balloon mitral valvotomy was performed in standard fashion using Accura balloon (Vascular Concepts Ltd., Halstead, UK) and considered successful when post-procedural MVA was >1.5 cm2, abolition of transmitral gradient (<5 mm Hg) and degree of mitral regurgitation < 2+ [17]. Tissue Doppler velocities and strain parameters were measured 24 hours after and three months later, following BMV in a manner similar to pre-procedure evaluation.

Statistical analysis

All statistical analysis was performed using SPSS Statistics v. 20 (IBM Corp., Armonk, NY). Numerical results were expressed as mean ± SD while categorical data were expressed as number and percentage. Pre- and post-BMV analyses were done using the paired t-test and Wilcoxon signed-rank test. The correlation between two variables was analyzed using the Pearson correlation coefficient. P-value < 0.05 was considered statistically significant.

## Results

Baseline parameters are shown in Table 1. Successful BMV was performed among 260 patients. Male and female were 109 (41.9%) and 151 (58.1%) respectively. The mean age was 26.7±4.7 years; 214 (82.3%) patients were in NSR while 46 (17.7%) had AF. Additionally, 16 (6.2%) patients had restenosis of the mitral valve, while 8(3.1%) patients had a prior history of stroke, of which 7 (88%) had permanent AF. Mean MVA was 0.89±0.11 cm^2^.

**Table 1:** Baseline characteristics of patients (N=260) NYHA: New York Heart Association; PASP: pulmonary artery systolic pressure; TR: tricuspid regurgitation; PHT: pressure half time

Variables	Number (Percentage)
Male/ Female	109 (41.9%) / 151 (58.1%)
Age distribution (years)	
a. Juvenile (below 18)	37 (14.2%)
b. 18-30	116 (44.6%)
c. 31-40	64 (24.6%)
d. 41-50	35 (13.5%)
e. Above 50	8 (3.1%)
Normal sinus rhythm	214 (82.3%)
Atrial fibrillation	46 (17.7%)
History of stroke	8 (3.1%)
NYHA functional class	III±0.6
Mitral valve area (cm^2^)	0.89±0.11
Mitral valve restenosis	16 (6.2%)
Mitral valve gradient (mmHg)	21.4±3.22
PASP (mmHg)	63.6±8.5
Peak TR gradient (mmHg)	52.4±3.8
PHT (millisec)	190±18

**Figure 2:**
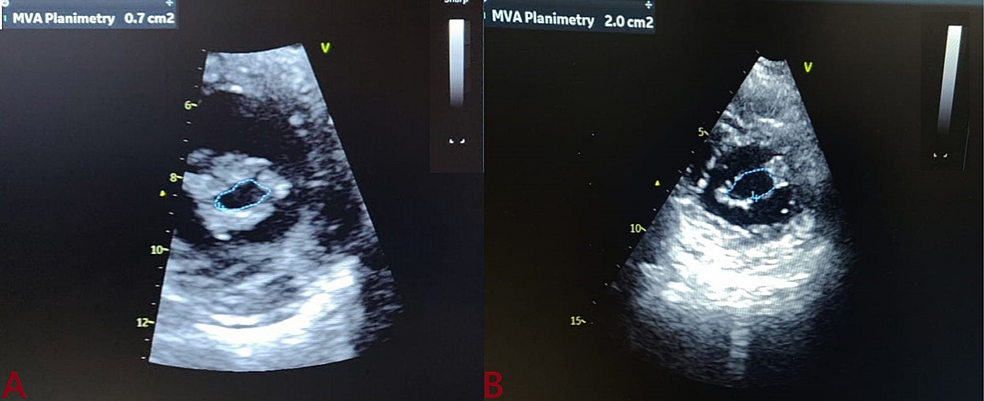
Change of valve area following BMV (A: baseline MVA; B: post-BMV). MVA: mitral valve area; BMV: post balloon mitral valvuloplasty

Following balloon dilatation, a significant increase in MVA was noted (0.89±0.11 cm^2^ vs. 1.83±0.3 cm^2^; p<0.001). PALS improved significantly immediately following the procedure (6.5±11.6% vs. 7.7±10.5%; p< 0.001) and continued to improve at three months of follow up (6.5±11.6% vs. 11.3±12.5%; p<0.001) which corresponded to 24% and 74% increase from baseline respectively. LA dimension also reduced immediately after BMV (42.4 ± 7.6 mm vs 41.1 ± 5.4 mm; p=0.076) and at three months of follow up a significant reduction was noted (42.4 ± 7.6 mm vs. 40.3±4.2 mm; p=0.043). Similarly, a significant reduction was observed in LA volume, and LA volume index (56.8±14.3 ml/m^2^ vs 48.4±12.5 ml/m^2^) immediately post BMV and (56.8±14.3 ml/m^2^ vs. 45.4±13.3 ml/m^2^) at three months of follow-up. Whereas MVA increased significantly post BMV from 0.89±0.11 cm^2^ to 1.83±0.3 cm^2^, and at three months after BMV MVA was 1.89±0.2 cm^2 ^(Figure 2). Similar pulmonary artery systolic pressure (PASP) decreased significantly after BMV (63.6±8.5 mmHg vs. 58.3±6.3 mmHg; P value-0.004) and (63.6±8.5 mmHg vs. 34.4±4.5 mmHg; P value-0.031) immediately and at three months after BMV respectively (Table 2). Patients with atrial fibrillation (AF) had a lesser improvement in PALS (60%) in comparison to those with the normal sinus rhythm (NSR) (60% vs. 84%; p=0.044) at three months. At three months, the increase in PALS was also less when compared to patients without a history of stroke (55% vs 80%; p=0.039) (Table 3). There was only an 8% reduction in PASP within 24 hours after BMV while the overall reduction was 45% at three months of follow-up. In comparison to this, a reverse trend was observed for the reduction in mitral valve gradient (MVG). Additionally, a 63% decrease in the MVG occurred immediately post BMV and an additional 7% decrease in MVG occurred over the next three months of follow-up.

**Table 2:** Echo/Doppler and left atrial strain parameters at baseline and following BMV (N=260) PALS: peak atrial longitudinal strain; LA: left atrium; MVA: mitral valvular area; MVG: mitral valve gradient; PASP: pulmonary artery systolic pressure; TR: tricuspid regurgitation; PHT: pressure half time; BMV: balloon mitral valvuloplasty; %: percentage P-values: p^a^=pre-BMV vs 24 hours post-BMV; p^b^=pre-BMV vs three months post-BMV; p^c^=24 hours post-BMV vs three months post-BMV.

	Pre BMV	24-hours post BMV	Average change (%) at 24 hours from baseline	3 Month Post BMV	Average change (%) at 3 months from baseline	P-value	p^a^	p^b^	p^c^
PALS (%)	6.5±11.6	7.7±10.5	+ 24%	11.3±12.5	+74%	<0.001	<0.001	<0.001	<0.001
M mode LA dimension (mm)	42.4 ± 7.6	41.1±5.4	- 3%	40.3±4.2	- 5%	0.043	0.076	0.049	0.066
LA volume (ml)	90.6 ± 31.1	66.6±18.7	- 26%	61.6±13.4	- 32%	<0.001	0.033	0.021	0.041
Indexed LA volume (ml/M^2)^	56.8±14.3	48.4±12.5	- 15%	45.4±13.3	- 20%	<0.001	0.043	0.021	0.047
MVA by planimetry (cm^2^)	0.89±0.11	1.83±0.3	+ 106%	1.89±0.2	+ 112%	<0.001	0.031	0.011	0.678
MVG (mmHg)	21.4±3.22	7.83±2.12	- 63%	6.34±1.8	- 70%	<0.001	0.031	0.023	0.044
PASP (mmHg)	63.6±8.5	58.3±6.3	- 8%	34.4±4.5	- 45%	<0.001	0.004	0.031	0.021
Peak TR gradient (mmHg)	52.4±3.8	49.2±3.3	- 6.1%	27.6±3.3	- 47.3%	<0.001	0.041	0.032	0.001
PHT (millisec)	190±18	170±12	- 10.5%	130±11	- 31.5%	0.002	0.059	0.034	0.078
M mode LV dimensions in diastole (mm)	39.7 ± 3.9	43.3±2.2	+ 9%	45.2±2.1	+ 13.8%	<0.001	<0.031	<0.021	0.056
M mode LV dimensions in systole (mm)	28.8 ± 3.9	29.3±2.2	+ 1.7%	31.3±2.3	+ 8.7%	0.435	0.743	0.347	0.789

**Table 3:** Increase in PALS in different sub-groups at three months PBMV. PALS: peak atrial longitudinal strain; PBMV: post balloon mitral valvotomy

Variables	Percentage
PALS increase at three-month follow-up in the whole study population	74%
PALS increase at three-month follow up among stroke patient	55%
PALS increase at three-month follow up among patient of atrial fibrillation	60%
PALS increase at three-month follow up in females older than 40 years of age	44%
PALS increase at three-month follow up in males younger than 30 years of age and having sinus rhythm	88%
PALS increase at three-month follow up in patients with normal sinus rhythm	80%

## Discussion

Our study demonstrated that the LA reservoir function assessed by PALS is abnormal in patients with mitral stenosis, which showed a significant increase within 24 hours after BMV, and this trend continued at three months of follow-up. Using the onset of atrial contraction as a reference may lead to a greater physiological strain curve, allow easier measurement of the contractile phase, and has been reported to be more predictive of outcome in patients with new-onset heart failure [18-20]. However, in our study end-diastole was taken as a reference point of LA strain measurement, which has an advantage as measurements could be obtained in all patients irrespective of rhythm and it also facilitated easy measurement of LA reservoir function as this reference equated well with a positive peak systolic value of the LA strain curve. Since there is no evidence demonstrating the superiority of one approach over the other, and given the disadvantage of the lack of onset of atrial contraction in atrial fibrillation/flutter, the end-diastolic time reference is commonly used for left atrial strain measurements world over.

The left atrium enlarges as a result of pressure and volume overload and undergoes remodeling in patients with mitral stenosis. A wide range of LA pressure exists in rheumatic MS despite similar MVA because an important determinant of LA pressure is LA compliance. Despite similar MVA, patients with rheumatic MS have a different grade of depressed left atrial compliance and hence different stiffness [21]. Abnormal LA stiffness may induce atrial fibrillation, intra atrial stasis with dense spontaneous contrast, and thrombogenesis.

The findings of our study, i.e. significant improvement of PALS, are in line with other studies published. Rohani et al. documented the acute effect of BMV and MVR in patients with mitral stenosis. Positive peak LA strain improved significantly after BMV [22].

Change in LA volume was concordant with findings reported by Rohani et al. [22] and Ansari et al. [23], and Vijayvergia et al. [24] although their findings were limited among patients with sinus rhythm only, while in our study, patients with both NSR and AF were recruited. Assessment of atrial function using atrial volume is operator- and load‑dependent and thus lacks accuracy. Its assessment using PALS is independent of rhythm and can better predict changes in atrial function following BMV [25].

In our study, left atrial volume and systolic strain were inversely related and helpful in the assessment of left atrial remodeling and predictive of atrial fibrillation, which was concordant with findings reported by Tsang et al. [26] who also reported that left atrial volume was a better and more sensitive predictor of AF and other cardiovascular events when compared to left atrial dimension. Thus left atrial strain parameters may also be helpful in assessing the left atrial remodeling and prediction of atrial fibrillation.

Regarding the reduction in trans-mitral mean pressure gradient and systolic pulmonary artery pressure, our findings were concordant with the report by Vijayvergia et al. [24]. Although these changes were quite evident following the procedure, these parameters continued to improve at three months after BMV, which indicates chronic long-term remodeling. 

It has been demonstrated that the left atrial dimension assessed by M mode doesn´t reduce significantly at 24 hours after BMV but reservoir function certainly changes when assessed using peak atrial longitudinal strain. This can be explained as changes in the LA dimension are the long-term change which follows, usually evident at the six-month follow-up, while reservoir function improved immediately following the procedure as shown in our study. This was coherent with findings reported by Ansari et al. [23] and Vijayvergiya et al. [24]. 

PALS is one of the best predictors for maintenance of sinus rhythm among patients with lone atrial fibrillation as reported by Di Salvo et al. [27]. They observed that patients with higher PALS have a greater likelihood of staying in sinus rhythm following successful cardioversion. Thus, improvement in PALS after BMV in our study may indicate their chance to continue to have sinus rhythm and lower cardiovascular events.

In a study by Liao et al., women had higher PALS than men, and aging deteriorates it in both sexes although more prominent among women in advanced age [28]. In our study, females older than 40 years showed the least increment (44%) in PALS while males who were younger than 30 years with sinus rhythm had the maximum increase in PALS at three months following BMV.

In our study, we documented that the successful BMV leads to the improvement of LA function, LA dimensions, and improvements in these parameters continue at three months of follow-up. The PALS is an important method to evaluate the LA reservoir function and it should be used in patients with mitral stenosis for decision-making before intervention and to see the effect of the intervention.

Limitations of the study 

We followed the patients only for three months; a longer follow-up would have given a better understanding of the effect of BMV on LA function in patients with mitral stenosis.

## Conclusions

The LA reservoir function can be better assessed by PALS. It is independent of rhythm and is better at predicting changes in atrial function. It is reduced in patients with severe mitral stenosis. PALS improves significantly within 24 hours following BMV, and improvement continues in the months following the procedure. PALS should be evaluated in patients with mitral stenosis for decision-making before intervention and to see the effect of the intervention. The improvement in PALS after BMV increases the probability of continuing to have sinus rhythm and lower cardiovascular events in the future.
